# AS03 Adjuvanted AH1N1 Vaccine Associated with an Abrupt Increase in the Incidence of Childhood Narcolepsy in Finland

**DOI:** 10.1371/journal.pone.0033536

**Published:** 2012-03-28

**Authors:** Hanna Nohynek, Jukka Jokinen, Markku Partinen, Outi Vaarala, Turkka Kirjavainen, Jonas Sundman, Sari-Leena Himanen, Christer Hublin, Ilkka Julkunen, Päivi Olsén, Outi Saarenpää-Heikkilä, Terhi Kilpi

**Affiliations:** 1 Department of Vaccines and Immune Protection, National Institute for Health and Welfare, Helsinki, Finland; 2 Helsinki Sleep Clinic, Vitalmed Research Centre, Helsinki, Finland; 3 Department of Pediatrics, Children's Hospital, Helsinki University Hospital, Helsinki, Finland; 4 Department of Clinical Neurophysiology, Tampere University Hospital, Tampere, Finland; 5 Finnish Institute of Occupational Health, Helsinki, Finland; 6 Department of Infectious Disease Surveillance and Control, National Institute for Health and Welfare, Helsinki, Finland; 7 Department of Child Neurology, Oulu University Hospital, Oulu, Finland; 8 Department of Pediatrics, Tampere University Hospital, Tampere, Finland; University of Hong Kong, Hong Kong

## Abstract

**Background:**

Narcolepsy is a chronic sleep disorder with strong genetic predisposition causing excessive daytime sleepiness and cataplexy. A sudden increase in childhood narcolepsy was observed in Finland soon after pandemic influenza epidemic and vaccination with ASO3-adjuvanted Pandemrix. No increase was observed in other age groups.

**Methods:**

Retrospective cohort study. From January 1, 2009 to December 31, 2010 we retrospectively followed the cohort of all children living in Finland and born from January 1991 through December 2005. Vaccination data of the whole population was obtained from primary health care databases. All new cases with assigned ICD-10 code of narcolepsy were identified and the medical records reviewed by two experts to classify the diagnosis of narcolepsy according to the Brighton collaboration criteria. Onset of narcolepsy was defined as the first documented contact to health care because of excessive daytime sleepiness. The primary follow-up period was restricted to August 15, 2010, the day before media attention on post-vaccination narcolepsy started.

**Findings:**

Vaccination coverage in the cohort was 75%. Of the 67 confirmed cases of narcolepsy, 46 vaccinated and 7 unvaccinated were included in the primary analysis. The incidence of narcolepsy was 9.0 in the vaccinated as compared to 0.7/100,000 person years in the unvaccinated individuals, the rate ratio being 12.7 (95% confidence interval 6.1–30.8). The vaccine-attributable risk of developing narcolepsy was 1∶16,000 vaccinated 4 to 19-year-olds (95% confidence interval 1∶13,000–1∶21,000).

**Conclusions:**

Pandemrix vaccine contributed to the onset of narcolepsy among those 4 to 19 years old during the pandemic influenza in 2009–2010 in Finland. Further studies are needed to determine whether this observation exists in other populations and to elucidate potential underlying immunological mechanism. The role of the adjuvant in particular warrants further research before drawing conclusions about the use of adjuvanted pandemic vaccines in the future.

## Introduction

To protect the population from death and serious forms of disease caused by the pandemic AH1N1 infection, the ASO3 adjuvanted vaccine Pandemrix was introduced nation-wide in Finland from October 2009 onwards according to the strategic prioritization order (Table 1) [1]. No other pandemic vaccines were available in the country. Vaccination was carried out as soon as the vaccines arrived in the country, starting 12th October 2009. Following recommendation of the European Medicines Agency (EMA), enhanced passive surveillance of vaccine related adverse events was initiated. Excess number of narcolepsy-cataplexy among children and adolescents was observed a few months after the A(H1N1) epidemic and pandemic vaccination [2]. Narcolepsy was not among the sentinel events EMA encouraged to be followed.

**Table 1:** The prioritization order of the pandemic influenza vaccinations in Finland during the A(H1N1) pandemic recommended by the National Advisory Committee on Vaccinations.

1.	Social and health care professionals who work with A(H1N1) infected patients or patients presumably exposed to the infection, as well as ambulance personnel, and pharmacists who work in customer service
2.	Pregnant women
3.	People aged 6 months to 64 years at high risk due to their underlying illness. This category includes persons who require regular medication for heart or lung disease, metabolic disease, chronic liver or kidney disease, immune deficiency because of an underlying condition or treatment, chronic neurological disease or neuromuscular disease
4.	Healthy children from 6 to 35 months of age
5.	Healthy children and adolescents from 3 to 24 years of age as well as army conscripts
6.	People aged 65 years and above who belong to high risk group due to an underlying illness. After this
7.	The rest of the population

Narcolepsy, a rare neurological sleep disorder characterized by excessive daytime sleepiness (EDS) and cataplexy, has never before the A(H1N1) pandemic been reported in association with vaccination [3], [4]. The cause of narcolepsy is unknown. Immunological mechanisms are considered instrumental to the onset of narcolepsy in genetically susceptible persons [5]–[7]. In addition, environmental factors capable of modulating immune system, e.g. streptococcal A and viral infections, have been suggested to trigger or accelerate disease development [6], [8]–[14].

To evaluate the observed safety signal suggesting association between Pandemrix vaccination and abrupt manifestation of narcolepsy in childhood and adolescence [1], [2], we first estimated the incidence of narcolepsy from register data and then performed a population based retrospective cohort study to verify the signal and to characterize its association with the pandemic vaccination.

## Methods

The study was done in Finland, a Northern European country with a population of 5.3 million and an annual birth cohort of approximately 60,000.

### Study population

The Finnish Population Information System, a computerised national register, allowed us to scrutinize the entire population. Personal data including name, gender, personal identity code, address, date of birth and death of all residents are recorded in this register. The personal identity code remains unchanged throughout a person's lifetime.

### Exposure to Pandemrix vaccination

**Figure 1:**
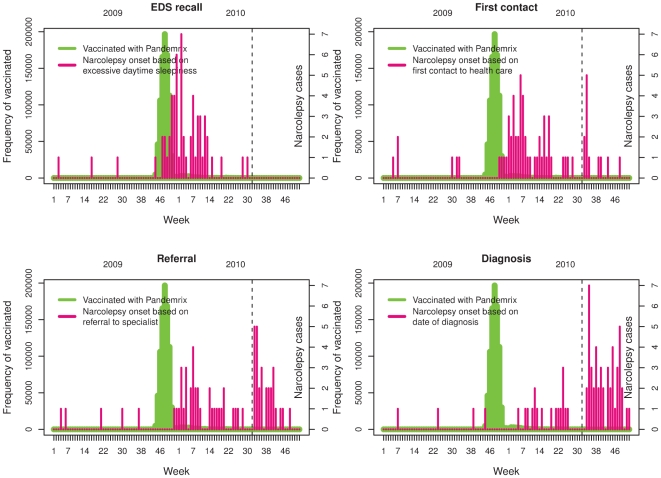
The temporal associations of pandemic vaccination, onset of narcolepsy (with four different definitions), and August 16, 2010, i.e. the date when the Swedish Medical Agency published the press release on the observation on the association between narcolepsy and Pandemrix vaccination (vertical dotted line). Panel top left is Recall = Parental/Patient recall when excessive daytime sleepiness (EDS) started; Panel top right is First contact = first contact to health care because of EDS; Panel bottom left is Referral = referral to specialist (paediatrician, neurologist); and Panel bottom right is Diagnosis = when diagnosis of narcolepsy was set.

Finnish municipalities (local governments) are responsible for the primary health care and subsequently the administration of the vaccines for the citizens in their region. Vaccinations with Pandemrix of those 19 years and below almost exclusively took place between weeks 44–52, 2009 (Figure 1), and were recorded in the electronic primary health care databases, which are linked to the Population Information System. Personal identity codes of the vaccinees and dates of vaccinations administered up till September 2010 were retrieved from these databases. The completeness of the exposure data was investigated by reviewing vaccination records of 1000 individuals that were randomly selected from the Population Information System.

### Screening of narcolepsy

Information on visits and hospitalizations assigned ICD-10 code G47.4 was obtained from the national care register covering all care provided in the Finnish hospitals for the years 1999–2009 and from the local hospital care registers for the year 2010. The same search was done in registers of the three specialized health care centers known to have the capacity of making the diagnosis of narcolepsy. The first recorded date was regarded as the date of diagnosis for that particular individual with narcolepsy. Incident cases of narcolepsy were calculated for the years 2009–2010 by using hereby determined dates of diagnosis assuming that if G47.4 was recorded for the first time in 2009 or later in the data representing years 1999–2010, it truly was the time when the diagnosis was set.

### Retrospective cohort study in the subgroup with increased incidence of narcolepsy

Having established that the increase in the incidence of narcolepsy occurred solely in the age group between 4–19 years [1], [2], we designed a retrospective cohort study of all children born during the period from January 1, 1991, to December 31, 2005 and living in Finland at any time during the years 2009–10. The primary follow-up period for this cohort started on January 1, 2009 and ended on August 15, 2010, the day before media attention on post-vaccination narcolepsy started in Finland.

Special attention was paid to case ascertainment and determining disease onset. All the relevant records of the ICD-10 G47.4-coded new patients belonging to the cohort and diagnosed during 2009–10 were reviewed [2]. Two narcolepsy experts (MP, TKir) independently reviewed the patient records and classified the cases according to the Brighton Collaboration criteria for diagnostic accuracy (Level 1, Level 2, Level 3, Unknown, or Not a case; work in progress www.brightoncollaboration.org, Table 2), The criteria are an extension of the American Academy of Sleep Medicine criteria for narcolepsy with added estimation of the reliability of the diagnosis. In the discrepant cases, the final level of diagnosis was set by a panel of three other narcolepsy experts (SLH, PO alternating with CH, OSH). A case was considered narcoleptic in the primary analysis, if it was classified as Level 1–3.

**Table 2:** Brighton collaboration criteria for diagnostic accuracy of narcolepsy.

Level	The Brighton collaboration criteria
**Level 1**	
In the *presence* of	
criterion 1	Excessive daytime sleepiness and/or definite cataplexy, AND
criterion 2	CSF hypocretin-1 deficiency
**Level 2**	
In the *presence* of	
criterion 1	Excessive daytime sleepiness, AND
criterion 2	Definite cataplexy, AND
criterion 3	Level 1 or 2 Multiple Sleep Test (MSLT) abormalities
**Level 3**	
In the *presence* of	
criterion 1	Excessive daytime sleepiness, AND
criterion 2	Level 1 MSLT abnormalities
In the *absence* of	Other mimicking disorders

In the primary analysis, the onset of narcolepsy was defined as the day when for the first time a school nurse, medical practitioner or other health care professional attended the patient because of the parental or own complaint of EDS, and recorded the observation in the patient records. This was considered the earliest objective time point available to define the onset time, and unlike the other time points available, less susceptible to the impact of media attention, which was initiated on August 16, 2010 by the press release on narcolepsy after Pandemrix vaccination given out by the Swedish Medical Authorities.

In the sensitivity analyses, three additional onset times were used, also to allow for comparison with earlier register data and with other studies.

#### Patient or parental recall

The two reviewers (MP, TKir) gave independent estimates of the onset time of symptoms (EDS and/or cataplexy) by reviewing the patient records. The patient or parental report of the time of onset usually had been recorded at the time of diagnostic workup. The mean date of these two estimates was used in the analysis.

#### Referral

The date of referral to a pediatrician or pediatric neurologist was the day when the attending clinician wrote a request of referral to a specialist.

#### Diagnosis

The date of diagnosis was defined as the date when the ICD code G47.4 was for the first time noted in the patient records.

### Statistical methods

The incidence of narcolepsy after exposure to H1N1 vaccination was compared to the incidence of narcolepsy without exposure to H1N1 vaccination using Poisson regression. Pandemic vaccination was treated as a time-dependent covariate meaning that subjects moved over from the unexposed state to the exposed state at the time of vaccination. Narcolepsy cases were grouped by vaccination status at the time of disease onset and the person times of the cohort in the vaccinated and unvaccinated states were used as weights in the analysis. The total person time in the cohort was calculated based on aggregate numbers of individuals by sex, year of birth, and region at the turn of 2009/2010 (immigration and emigration in the age group 4 to 19-year-olds in Finland is less than 0.3%). Person time in the vaccinated state was calculated based on weekly cumulative aggregates of the vaccinated during the follow-up. The results are expressed as the rate ratio with 95% confidence intervals based on profile likelihood. The relative rate was calculated by comparing incidences in the vaccinated and unvaccinated states during the follow-up in question. Absolute incidences were calculated by number of narcolepsy cases divided by the person times in the population in the respective states (vaccinated/unvaccinated). The vaccine attributable risk was calculated as the cumulative incidence in the vaccinated minus the expected cumulative incidence without vaccination during the same follow-up time.

In the primary analysis, the date of first contact to health care was used to pinpoint disease onset, and the follow-up time was from January 1st 2009 until August 15th 2010. The follow-up in the primary analysis started 10 months prior to the vaccination campaign. This was done in order to obtain information about the baseline incidence and to aqcuire more power to estimate the risk in the unvaccinated. Several sensitivity analyses using different onset definitions and follow-up times were conducted to investigate changes in the risk of the unvaccinated in calendar time, and bias potentially introduced by the increasing awareness among the health care workers and the public of the suspicion that there was a link between Pandemrix and narcolepsy. To minimize potential detection bias, follow-up periods ending as early as February 22, 2010 were also tested. This was the date when one of the authors (MP) for the first time raised the question of the association of one of the cases and H1N1 infection in a discussion between colleagues.

### Ethics statement

The study protocol was reviewed and approved by the Institutional Review Board of the National Institute for Health and Welfare (THL), Finland.

## Results

### Vaccination coverage in the population

In total, 2,76 million Pandemrix vaccine doses were given between October 2009 and August 2010. Vaccination coverage across the country was 52%, but varied from 32 to 82% in the different age groups (Table 3). In contrast, the geographical variability measured as variability across the 21 hospital districts of the country was low, particularly in children and adolescents ranging from 64 to 81%. Of the 915,854 individuals born between 1991 and 2005, 688,566 (75%) were vaccinated. All vaccinated individuals had received only one dose as recommended. The review of the vaccination records of the randomly selected 1000 individuals belonging to the study cohort revealed discrepancy between the local health care records and the electronic register data in four cases, all of whom had been vaccinated according to record review but not according to the database search. In addition to the sample of 1000, the vaccination records of all newly diagnosed narcolepsy cases born between 1991 and 2009 were also reviewed. No discrepancies were found.

**Table 3:** The age-specific Pandemic vaccination coverage in Finland during the influenza pandemic season in 2009–10.

Age group	N vaccinated^1^	N total^2^	Percentage
**0–4**	221,297	298,114	74.2
**5–9**	232,023	287,786	80.6
**10–14**	247,720	302,423	81.9
**15–19**	189,247	334,636	56.6
**20–24**	104,535	324,472	32.2
**25–29**	109,387	344,634	31.7
**30–34**	133,026	337,970	39.4
**35–39**	130,096	310,768	41.9
**40–44**	149,077	358,754	41.6
**45–49**	160,040	378,341	42.3
**50–54**	168,853	378,037	44.7
**55–59**	189,854	388,165	48.9
**60–64**	220,640	396,886	55.6
**65–69**	149,071	258,319	57.7
**70–74**	131,876	225,043	58.6
**75–79**	101,793	179,671	56.7
**80-**	122,791	247,408	49.6
**Total**	2,761,326	5,351,427	51.6

Sources:

1 Electronic patient records in Finnish health care centres.

2 Population register of Finland;

### Patients with confirmed diagnosis in the retrospective cohort

Altogether 71 new diagnoses of narcolepsy were set in children and adolescents aged 4 to 19 years of age in 2009–10 according to the G47.4 ICD10 code. Medical records were obtained from all. Based on the expert review of the hospital and primary care records, the diagnosis of narcolepsy was classified as being level 1 in 11 (16%), level 2 in 51 (76%), and level 3 in 5 (8%) of the patients according to the Brighton collaboration definitions. The two reviewers differed in their opinion on level of classification in three cases. In full agreement by the reviewers, four cases were classified as unknown or not a case. Of the 67 confirmed cases, 57 (85%) sought medical care and 61 (91%) received the diagnosis after pandemic vaccination. Thirty-three were female, 34 male. A detailed clinical description of the patients constituting most of the cohort of narcoleptic cases seen in 2010 has been provided elsewhere [2].

**Figure 2:**
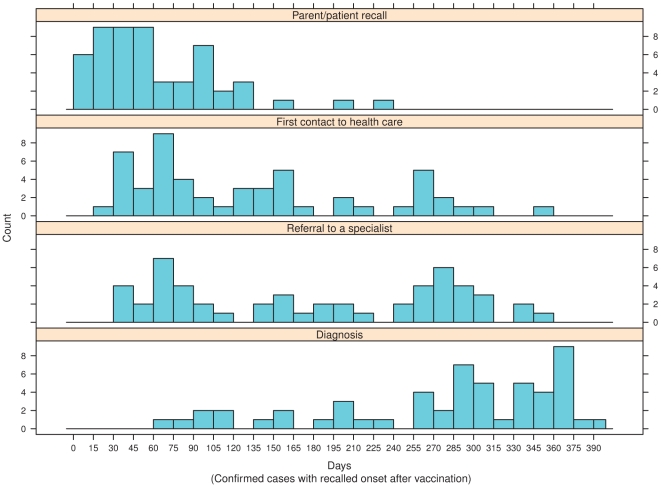
The different time intervals from the vaccination to the onset of narcolepsy depending on the definition of the onset time point, i.e. a) estimated onset time based on the extensive review of the patient records by a sleep and/or narcolepsy specialist, and closest to the parental/patient recall; b) first recorded contact to health care because of excessive sleepiness; c) date of referral to paediatrician or pediatric neurologist; and d) date of setting the diagnosis of narcolepsy, ICD-10 G47.4.

**Figure 3:**
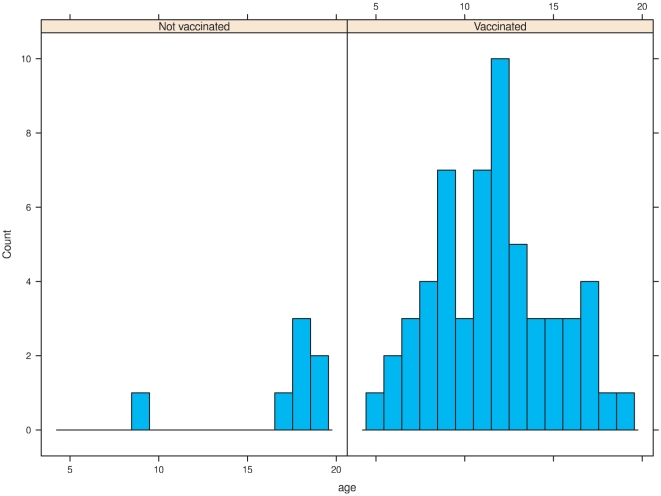
The age distribution of the new narcoleptic cases among the Pandemrix vaccinated and unvaccinated children and adolescents. Age presented in years.

Twenty of the first health care contacts were documented in school medical records, 21 in health centres, 8 in private practice, and the rest in hospitals. The time elapsed from vaccination to the onset of disease varied depending on the definition used for onset (Figure 2). Eighteen children were referred to a specialist already after Christmas 2009 and prior to the end of February 2010, 15 children were referred between 1 March 2010 to 15 August 2010, prior to the media attention, and 27 on or shortly after this date (Figure 2). The effect of the media attention shows as a bimodal distribution of the date of the first contact and referral (Figure 1, panels top right and bottom left; Figure 2). The delay from referral to the diagnosis was generally shorter for those referred on or after August 16, 2010 than before (mean delay 42 vs.122 days). The vaccinated patients were younger than those unvaccinated (Figure 3). Geographically, cases occurred in 16/21 Finnish hospital districts. This is in accordance with the underlying population size.

**Figure 4:**
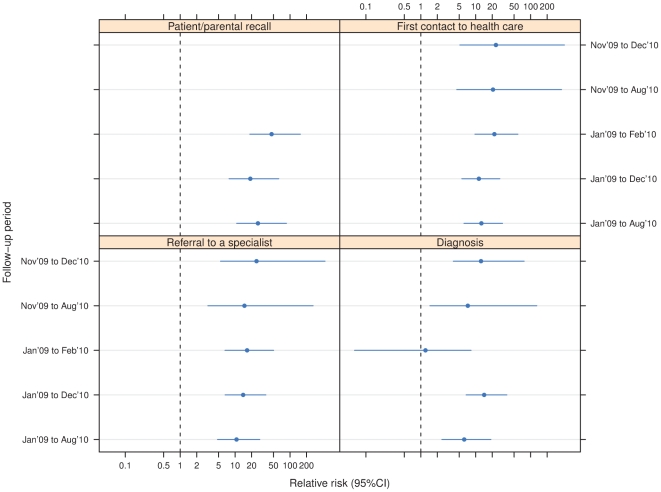
Sensitivity analyses of the risk ratio of Pandemrix vaccination and narcolepsy using different definitions of the onset dates of narcolepsy and follow-up time periods. The two intervals in the top left panel are missing because of infinite estimates (i.e. no cases among unvaccinated).

In the primary analysis, the incidence of narcolepsy was 9.0 in the vaccinated as compared to 0.7/100,000 person years in the unvaccinated children and adolescents, translating into a rate ratio of 12.7 (95% confidence interval 6.1–30.8) (Table 4). The lower limit of the 95% confidence level of the rate ratio was well above one in all sensitivity analyses using different follow-up periods and onset time definitions, except for the date of diagnosis as onset definition and follow-up period ending February 22, 2010 (Figure 4).

**Table 4:** Main results of the cohort analysis using two follow-up periods among those born at or after 1 January 1991.

Incidence in confirmed narcolepsy cases							
Follow-up period	Narcolepsy cases		Follow-up years		Relative Risk		
	Not vaccinated	Vaccinated	Not vaccinated	Vaccinated	Risk ratio	95%LCL	95%UCL
First contact:2009-01-01 to2010-12-31	7	57	1,069,247	762,461	11.4	5.6	27.5
First contact:2009-01-01 to 2010-08-16^1^	7	46	986,195	510,874	12.7	6.1	30.8

1 The date when the news on the possible association between narcolepsy and Pandemrix vaccination observed in Sweden was published in the national media in Finland.

LCL = Lower confidence limit, UCL = Upper confidence limit.

Six cases of narcolepsy had their first health care contact prior to the first H1N1 epidemic and the vaccination campaign. During the prepandemic and prevaccination follow-up period from January to October 2009, the baseline incidence of narcolepsy in the age-group of 4–19-year-olds was estimated as 0.79/100,000 person-years. No obvious change in the rate of unvaccinated was observed after the start of the campaign: By the time of media attention in August 2010, one case was recorded in the 227,288 unvaccinated, compared to an expected of 1.8 cases. With the estimated incidence in the vaccinated (9.0/100,000 person-years), one would have expected 20.6 unvaccinated cases.

Based on the primary analysis, the vaccine attributable risk of developing narcolepsy within approximately 8 months after Pandemrix vaccination was estimated to be 1 in 16,000, with 95% confidence interval from 1 in 13,000 to 1 in 21,000 vaccinated.

## Discussion

We found a 12.7-fold risk of narcolepsy in 4–19-year-old individuals within approximately 8 months after Pandemrix vaccination as compared to unvaccinated individuals in the same age group. This translates into a vaccine attributable risk of 1∶16,000.

Our study covers the entire population of Finland and is based on comprehensive data on individual Pandemrix vaccinations, diagnoses of narcolepsy and linkage of the two using unique personal identification codes assigned to all residents in Finland. Vaccination records were retrieved from primary health care databases. The high accuracy of the exposure data was confirmed through a validation check on a random sample. Newly diagnosed cases of narcolepsy were identified via a systematic nationwide search from the hospital registers, and the diagnoses were verified through a systematic stepwise expert review procedure.

Some parents may have been tempted to recall the onset of symptoms as occurring after, rather than before their child received the pandemic vaccine. Therefore, we used different definitions for disease onset to evaluate the significance of the timing of onset on the observed association. In the primary analysis, the earliest note of EDS in the patient's medical records was used to limit recall bias.

A particular concern is that the observed association is a result of increased detection of narcolepsy among vaccinated children. According to such a view, a similar increase in narcolepsy among unvaccinated children has occurred but is yet to be observed. This argument, however, is not supported by the factual circumstances. In early 2010, narcolepsy was a rare disease unknown to most parents. Also, very few primary care physicians had seen a narcoleptic child, and no beliefs, even less conviction associated narcolepsy with the pandemic vaccine. Yet considerable numbers of Pandemrix vaccinated children were already referred to specialist before the end of February 2010 and later diagnosed with narcolepsy. The sudden surge of referrals during the first months of 2010 can hardly be explained by increased awareness and changes in diagnostic practices alone. Awareness was aroused and referrals to specialist and diagnostic workup expedited only after the media attention from Sweden broke out in August 2010.

Should a confounding factor instead of vaccination be the true cause of the association, it would have to be even more strongly associated with narcolepsy than the pandemic vaccination as we now report. In addition, such a risk factor should have a strong and time dependent positive correlation with the vaccination itself. A recent study in China found a 3–4-fold greater than predicted occurrence of narcolepsy onset following the 2009–10 H1N1 pandemic season, which was independent of vaccination [14]. In our study, there was no evidence of change in the incidence among the unvaccinated 4–19-year-olds after the first H1N1 epidemic in Finland, whereas a considerably increased risk was associated with vaccination. As H1N1 infection was hardly more common in the vaccinated than in the unvaccinated population, our findings contradict the Chinese observation. We can think of several infectious, environmental, social or psychological factors that could modify the strength of the association seen in this study but none that could completely undo an association of this magnitude.

Our finding is supported by the recent results from Sweden, where a cohort study covering the entire population reported an almost 7-fold incidence of narcolepsy with cataplexy in children vaccinated with Pandemrix compared to those in the same age group who were not vaccinated [4]. The incidence in the unvaccinated (0.64/100,000 person-years) compares well to that seen in our study. Preliminary passive reporting system data from France, Norway and Ireland also indicate higher than expected number of cases in children and adolescents after Pandemrix vaccination [15]–[17]. On the other hand, it is perplexing that both Canada and the United Kingdom lack the signal. In these two countries, genetic susceptibility to narcolepsy is as common as in the Nordic countries. This suggests multifactorial nature of the observed phenomenon.

The biological plausibility for a vaccine contributing to the increased risk of narcolepsy particularly in the signal-generating age group is based firstly on the immunomodulatory effects of vaccination and secondly on the fact that narcolepsy is strongly linked to the HLA DQB1*0602 allele [18]. An analogous example of a similar disease process affecting children and adolescents in particular is provided by type 1 diabetes, in which insulin-producing beta-cells are destroyed by immunological mechanisms in genetically predisposed individuals with HLA DQB1*0302 and 02 alleles [19]–[21]. Neither an increase nor an imbalance between the vaccinated and unvaccinated in the incidence of narcolepsy was seen in the population older than 19 years [2]. It is noteworthy that the HLA DQB1*0602 allele is approximately twice as common in northern than in southern Europe [22] and that apart from the Nordic countries, Ireland and Canada, the AS03 adjuvanted vaccine was not widely used in the age group from 4 to 19 years. It should therefore not be surprising that the signal was detected in Sweden and Finland.

Vaccinations may induce bystander activation of immunological responses especially due to function of adjuvants. The age-related differences in the immune responsiveness to Pandemrix vaccination may be of importance in the induction of the bystander activation of immune system [23]. Pandemrix vaccination could have accelerated an on-going disease process rather than triggered narcolepsy associated autoimmunity. As computer search for peptide homologies between H1N1 virus and neuron-specific proteins did not reveal any potential molecular mimicry [7], [24]–[27], it seems unlikely that H1N1 virus infection or vaccination induced cross-reactive autoimmunity against hypocretine-producing neurons.

Our finding raises concerns of lipid containing adjuvants. Animal models have suggested that squalene, although at higher doses than used in human vaccines, is capable of contributing to the development of autoimmunity [28]–[30]. In humans, the epidemiological data available until now has not supported the induction of autoimmunity by squalene containing adjuvants. Adjuvanted vaccines are much needed to enhance immune responses, especially in immune compromised persons. The large scale use of new adjuvanted vaccines in human populations calls for further research of their association with adverse effects, such as autoimmunity.

Further studies are urgently needed to determine whether the association between adjuvanted pandemic vaccinations and narcolepsy can be demonstrated in other populations. The underlying immunological mechanism also warrants further research.
